# Re-imagining Health Research Partnership in a Post-COVID World: A Response to Recent Commentaries

**DOI:** 10.34172/ijhpm.2020.69

**Published:** 2020-05-12

**Authors:** Sarah Bowen, Ingrid Botting, Ian D. Graham, The Building and Managing Effective Partnerships in Canadian Health Research Research Advisory Team

**Affiliations:** ^1^Applied Research and Evaluation Consultant, Centreville, NS, Canada.; ^2^University of Manitoba, Winnipeg, MB, Canada.; ^3^University of Ottawa, Ottawa, ON, Canada.

If there was ever a time to rethink many of the research paradigms under which we have been operating,^1^ it is at this time of global upheaval as the world continues to struggle with the coronavirus disease 2019 (COVID-19) pandemic. At the time our research “*Experience of health leadership in partnering with university-based researchers in Canada – a call to ‘re-imagine’ research”* was published,^2^ we did not know to what extent experiences with research partnerships reported by many in Canada would resonate with a broader audience, or in other areas of the world. Commentaries from experts working with many different health systems indicate that, unfortunately, the challenges we reported are not unique to Canada. At the same time, we were deeply encouraged by the insights offered by these commentators, which both broadened and deepened the conversation we had hoped to initiate through this publication.

Along with much shared experience around the challenges of research-health system partnerships, there appears to be broad consensus on a number of issues: the need to reframe and reorient how we think about research^3-8^; the values and benefits of research co-creation^1,9^; the role of mutual learning^6,10^; the importance of responding appropriately to complexity^1,4,7,8,11^; and the need to move from ‘one-off’ projects to long term relationships.^1,4,6,9,10^

Given the level of consensus on key characteristics of the challenge ahead of us, it is time to move from discussion of challenges to action to address them. Many commentators outlined examples of innovative strategies of potential benefit to many other jurisdictions.^3-6,10^ Our research did not explore the range of possible responses to the identified challenges, and we hope that the examples provided – of strategies at the individual, group, organizational and system levels - will stimulate further discussion and greater sharing: there is much to be learned from the experience of other jurisdictions and alternate approaches.

It is indeed time for action: this action should focus on changes at the *systems* level. Changes are needed in how we conceptualize and integrate research into action; in how the various worlds of academia, government, and health services interact with each other. While evidence-informed innovations to increase individual and organizational capacity to engage in and manage research should be supported, it is of greater urgency to address the structural and system issues underlying how we conceptualize, organize and fund research, including how we prepare researchers and health systems to effectively work together in addressing the critical issues of our time.

But action is not enough. Many of the initiatives promoted by participants in our study did not appear to be evaluated: it is essential that we integrate both evaluative thinking and evaluation expertise into all initiatives. Researchers are not always good evaluators and health systems too often do not integrate comprehensive evaluation strategies into implementation and assessment of initiatives.^12^ Valuing the role of evaluation in health service innovation is one critical strategy for bridging the research/evaluation/quality improvement divide and demonstrating the humility required for mutual learning. Thoughtful evaluation will help to ensure that we can identify principles that may be transferable to other contexts: to know what works in what context.^6^ Adoption of developmental evaluation approaches can support both an initiative, and the organization supporting it, in mutual and ongoing action learning as initiatives are introduced, adapted, and changed.

But we need to be much bolder.^8^ It is not the time to be satisfied with well-intentioned efforts to tweak current research processes. Many are coming to terms with the frightening reality that things may never be the same again. At the same time, the COVID-19 crisis has presented opportunities, through challenging our assumptions and ways of working, to make the changes so desperately needed to restructure the conduct of research. There are many things we have learned through struggling to address this pandemic:

- *We can, and must, let go of common research myths that discourage bold action.* No, we do not need 17 years to get research acted on; we can change things now. Working together to address an identified problem (eg, rapid reviews conducted by COVID-19 synthesis teams around the world) does not mean that good research results take longer – effective partnerships can help us get there sooner, and are more likely to result in implementation of research findings.
- *All kinds of research are essential, and most important research issues are complex, requiring interdisciplinary responses.* Nothing researchers do in their own areas is adequate without the insights and expertise of other perspectives. Discovery research should be funded independently of current political priorities, and reflect evidence on global health priorities. And while discovery research is essential, research on applied solutions must receive greater support. We need clinical research to ensure better and safer treatments that respond to both healthcare realities and the life conditions and priorities of patients. We need epidemiologists to help understand and predict the impact of various choices. We need communication experts to help frame research evidence into messages that will help politicians, healthcare leaders, point-of-care providers, and the general public better understand the challenges before us (and these are several, not one, skill sets). But more than this: we need interdisciplinary research *teams* in order to appropriately address the ‘wicked’ problems of our times.^7^ Nor is research the domain of researchers alone: we need innovative strategies to ensure that non-researchers are valued members of research teams.
- *We need to continue to challenge the old models that separate ‘research’ from the important work of healthcare provision,* integrating research *expertise* into all aspects of health system decision-making. This will require creative rethinking of how researchers work with (and sometimes within) the system, and with communities and patients. We need to support authentic learning within healthcare organizations.
- *We must adequately resource what we say we value.* Health and social care systems must be adequately resourced so that they have the capacity to deliver quality care – not only in ‘normal times,’ but when faced by the unexpected and unimaginable. There must be adequate ‘slack’ within these systems to enable surge capacity: short-sighted cost savings may cost us more in the long run. Health systems must also have the capacity to adequately resource appropriate implementation of research findings – implementation can no longer be thought of as something that just happens. If we value research and believe that it should inform healthcare decision-making, we must ensure not only that there is funding for specific projects, but appropriate infrastructure to support ongoing health system research partnerships.
- *We need our research systems (and researchers) to be nimble.* As point-of-care providers know, it is necessary to use the evidence we have now because we need to act now. This is not to say that longitudinal studies or the rigour of randomized clinical trials are not also essential – but, as has been so clearly demonstrated over the past months, point-of-care staff need to make decisions while this research is still taking place. Over the past few months we have seen that we can fund and support ‘real-time’ research – we can apply this learning to other health issues when this crisis is over. Established research partnerships are needed if researchers are to be best positioned to help inform decision-making in real time.
- *All research should consider questions of equity.* As demonstrated through this pandemic, there is a complex interplay between different vulnerabilities and advantages – this intersectionality means not only that some individuals face greater risks of specific conditions, but health services work more or less well for different people.^13^ A commitment to equity also requires a broadening of our understanding of who should be ‘research partners.’We need effective strategies for partnerships not only with healthcare leadership and providers, but also with those with lived experience, and with providers whose voices often fail to reach decision-makers. How many opportunities for improved healthcare organization were missed, how many lives were lost, because of lack of inclusion of the insights of personal care workers, an invisible and voiceless group within most current health research?
- *Without confident, open relationships between researchers, health leadership, and political leaders, bad things will happen.* The scientific community must engage, with humility,^7^ with decision-making processes. Lack of confident, mutually respectful relationships (that recognize the specialized skills and insights of all partners), and failure to ensure good communication between scientists, healthcare providers, health leadership, and politicians can result in disaster.
- *We all need greater humility.* As noted in a recent post from the Executive Director of the Canadian Association of Health Services and Policy Research:

*“… humility is key to overcoming this virus. No one is a COVID-19 expert and mistakes will be made. Hypotheses will be wrong. Models are not crystal balls. Accepting that we are all learning together, being willing to change our thinking as new evidence comes to the fore and working from the same starting place has democratized the process for many.”* ^14^

Partnership is needed not only to solve problems, but – hopefully to avoid them. We can only echo the hope that the sharing of ideas and solutions, *“this democratization of the scientific process will extend past the COVID-19 pandemic and benefit other dilemmas we face in healthcare and beyond.”*^14^

## Acknowledgements

IDG is a recipient of a CIHR Foundation Grant (FDN# 143237). “*The Building and Managing Effective Partnerships in Canadian Health Research”* research team reviewed the commentaries and provided input into framing the response. Members of the research/advisory team include Ingrid Botting (PI), Ian Graham, Martha MacLeod, Karen Harlos, Danielle de Moissac, Christine Cassidy, Bernard Leduc, Cathy Ulrich, and Janet Knox.

## Ethical issues

Not applicable.

## Competing interests

Authors declare that they have no competing interests.

## Authors’ contributions

SB, IB, and IDG conceptualized the article, and the “*Building and Managing Effective Partnerships in Canadian Health Research”* research team provided input into framing the response. SB drafted the article with IB and IDG critically reviewing and editing it. All team members reviewed and approved the final version of the article and accept responsibility for it.

## Funding

Original research was supported by CIHR Foundation Grant (FDN# 143237).

## Authors’ affiliations

^1^Applied Research and Evaluation Consultant, Centreville, NS, Canada. ^2^University of Manitoba, Winnipeg, MB, Canada. ^3^University of Ottawa, Ottawa, ON, Canada.
